# The Effect of A2E on the Ca^2+^-PKC Signaling Pathway in Human RPE Cells Exposed to Blue Light

**DOI:** 10.1155/2022/2233223

**Published:** 2022-10-18

**Authors:** Maomei Luo, Shu Wang, Yun Tang, Chun Zeng, Shanjun Cai

**Affiliations:** ^1^Department of Ophthalmology, Guizhou Eye Hospital, Affiliated Hospital of Zunyi Medical University, Zunyi, Guizhou, China; ^2^Department of Ophthalmology, Dazhou Central Hospital, Dazhou, Sichuan, China; ^3^Zunyi Medical University, Zunyi, Guizhou, China

## Abstract

**Aims:**

In a model of blue light-induced damage in N-retinylidene-N-retinylethanolamine (A2E)-loaded human retinal pigment epithelial (RPE) cells, we examined the effect of A2E on the calcium (Ca^2+^)-protein kinase C (PKC) signaling pathway.

**Methods:**

Primary human RPE cells were cultured, and the cells in the 4th–6th passages were used in this study. The cells were divided into 5 groups: control cells (no A2E, no blue light), blue light-treated cells, blue light + chloroquine-treated cells, blue light + A2E-treated cells, and blue light + A2E + chloroquine-treated cells. The cells were first treated with chloroquine (15 *μ*M for 12 h) and then loaded with A2E (25 *μ*M for 2 h).The blue light intensity was 2000 ± 500 lux, and the duration was 6 h. After blue light exposure, the cells were cultured for 24 h. Fluo-3/AM staining was used to determine the level of cytoplasmic Ca^2+^, and the cells were photographed using a laser scanning confocal microscope to analyze the fluorescence intensity. The intracellular levels of inositol triphosphate (IP3) and diacylglycerol (DAG) were measured by enzyme-linked immunosorbent assay (ELISA). Intracellular PKC activity was measured with a nonradioactive nuclide assay.

**Results:**

Among all cell groups, the levels of Ca^2+^, DAG, and IP3 were lowest in the control cells (*P* < 0.05). The Ca^2+^, DAG, and IP3 levels in the blue light + A2E-treated cells and blue light + chloroquine-treated cells were higher than those in the blue light-treated cells (*P* < 0.05). The Ca^2+^, DAG, and IP3 levels were highest in the blue light + A2E + chloroquine-treated group (*P* < 0.05). PKC activity was lowest in the control cells (*P* < 0.05). The PKC activity of the blue light + A2E-treated cells and blue light + chloroquine-treated cells was higher than that of the blue light-treated cells (*P* < 0.05), and the PKC activity of the blue light + A2E + chloroquine-treated cells was the highest (*P* < 0.05).

**Conclusion:**

Blue light and A2E increased the levels of Ca^2+^, IP3, and DAG in human RPE cells and enhanced PKC activity, and blue light and A2E had a synergistic effect. Chloroquine further increased the levels of Ca^2+^, IP3, and DAG and PKC activity in RPE cells or A2E-loaded RPE cells exposed to blue light.

## 1. Background

Retinal pigment epithelial (RPE) cells are an important part of the blood–retinal barrier. These cells maintain retinal homeostasis by filtering damaging light, replenishing 11-cis retinal pigment clusters in photoreceptors, and engulfing the outer disc membrane of receptors. RPE lipofuscin is a byproduct of the phagocytosis of lipid-rich photoreceptor outer segments, and the major fluorophore is N-retinylidene-N-retinylethanolamine (A2E), which increases with age and is not easily degraded [1]. When A2E is irradiated by visible light or ultraviolet light, its structure changes, and free radicals such as superoxide anions, singlet oxygen, and hydrogen peroxide are produced; these free radicals can damage the structure of the cells [2].

In modern society, mobile phones, tablets, and computers emit high-energy blue light. The most harmful component of visible light is blue light (400–500 nm) [3]. Blue light can damage the function of RPE cells and reduce the reaction of photoreceptors to light. Toxic substances (e.g., A2E) produced by blue light irradiation are related to the development of degenerative diseases, such as age-related macular degeneration (AMD) and Stargardt's disease [4, 5].

Calcium (Ca^2+^) is an important second messenger in cells. Extracellular free Ca^2+^ levels are much higher than those within the cell, and more than 90% of Ca^2+^ is found in intracellular Ca^2+^ stores [6]. A rapid increase in the Ca^2+^ concentration in the plasma membrane or intracellular Ca^2+^ stores can trigger a chain reaction and a series of physiological effects. Protein kinase C (PKC), a target molecule of Ca^2+^ in the cell, is usually present in its inactive form. When extracellular signals activate phospholipase C (PLC) through receptors and decompose membrane phospholipids, inositol triphosphate (IP3) and diacylglycerol (DAG) are produced [7]. IP3 promotes the release of Ca^2+^ from Ca^2+^ stores, resulting in an increase in the intracellular Ca^2+^ concentration, which activates PKC.

Therefore, we utilized the autophagy function of lysosomes and the effect of chloroquine on lysosomal stability to explore the phototoxic effects of A2E on intracellular Ca^2+^, IP3, and DAG levels and PKC activity in the Ca^2+^-PKC signaling pathway during blue light irradiation.

## 2. Methods

### 2.1. Cell Sources and Ethics Statement

Human primary RPE cells were cultured, and three donor eyeballs were obtained after corneal transplantation in the Department of Ophthalmology, Affiliated Hospital of Zunyi Medical University. All experiments were approved by the Ethics Committee of Zunyi Medical University, China ([2017]1–111).

### 2.2. Reagents and Instruments

Dulbecco's modified Eagle's medium (DMEM), fetal bovine serum (FBS), and a trypsin-EDTA solution (0.25%) were purchased from Gibco (Logan, UT, USA). Penicillin–streptomycin was purchased from HyClone (Logan, UT, USA). Fluo-3/AM was purchased from Thermo Scientific (Waltham, MA, USA). Enzyme-linked immunosorbent assay (ELISA) kits were purchased from Jianglai Biotechnology (Baoshan, Shanghai, China). A PKC kinase activity assay kit was purchased from Promega (Madison, WI, USA). A2E was donated by the First Affiliated Hospital of Shanghai Jiaotong University.

### 2.3. Primary Human RPE Cell Culture and Treatment

Briefly, the eyeballs were cut approximately 5-6 mm behind the corneal limbus, and the anterior segment, vitreous body, and retinal neuroepithelial layer were carefully removed to make an eye cup, which was then washed with HBSS. Approximately 2/3 of the eye cup was filled with 0.25% trypsin, and the eyeballs were incubated at 37°C for 30 min. DMEM containing 10% FBS was used to stop the reaction, and the cell suspension was collected and centrifuged at 1000 r/min for 8 min. The precipitate was resuspended in DMEM supplemented with 15% FBS, and the cells were cultured in a 25 cm^2^ cell culture flask at a density of 3 × 10^4^ cells/ml. When the cells reached approximately 80% confluence, they were passaged at a dilution of 1 : 2. Cells from the same passage were used for each experiment. Cells cultured in DMEM without other treatments were considered control cells. Cells illuminated with blue light were named blue light-treated cells. Cells that were exposed to blue light and chloroquine, A2E, or chloroquine plus A2E were named blue light + chloroquine-treated cells, blue light + A2E-treated cells, and blue light + *A*2E + chloroquine-treated cells, respectively. Chloroquine at a final concentration of 15 *μ*M was added to the cells and incubated for 12 h. Then, 25 *μ*M A2E was added to the cells and incubated in the dark for 2 h before the cells were illuminated [8]. The blue light intensity was 2000 ± 500 lux, and the duration was 6 h; illumination was followed by culture for 24 h [9].

### 2.4. Immunofluorescence Analysis

RPE cells were fixed for 10 min with 4% paraformaldehyde. After being rinsed in PBS, the cells were blocked with 1% Triton X-100 and 10% goat serum at room temperature for 30 min, followed by incubation with mouse anti-human RPE65 antibodies overnight at 4°C and goat anti-mouse IgG secondary antibodies for 1 h at 37°C. 4′,6-Diamidino-2-phenylindole (DAPI) was used for nuclear staining. Immunoreactive cells were visualized, and images were recorded using an inverted confocal microscope.

### 2.5. Intracellular Ca^2+^ Detection

Ca^2+^ levels were measured using the fluorescent probe Fluo-3/AM. Briefly, Fluo-3/AM was dissolved in DMSO and diluted with HBSS to a final concentration of 5 *μ*M in the dark. After treatment, the cells were washed and stained with Fluo-3/AM for 45 min at 37°C in the dark. The fluorescence intensity of Ca^2+^ was quantified with a laser scanning confocal microscope at an excitation wavelength of 488 nm and an emission wavelength of 525 nm.

### 2.6. Determination of Intracellular IP3 and DAG Levels

The cells were grouped and pretreated as described in Section 2.5. The supernatants and cells were collected, and the levels of IP3 and DAG were measured using commercial ELISA kits according to the manufacturer's instructions. The absorbance at 450 nm was measured with a microplate reader.

### 2.7. Determination of Intracellular PKC Activity

PKC activity was measured using a PKC activity assay kit according to the manufacturer's protocol. Cells were homogenized and centrifuged to isolate the proteins, a cellulose column was used to separate the PKC fraction, and an agarose gel was used for electrophoresis to separate the two different PKC proteins (phosphorylated PKC and nonphosphorylated PKC). The ratio of the gray value of phosphorylated PKC to that of nonphosphorylated PKC indicated the level of PKC activity (PKC activation).

### 2.8. Statistical Analysis

The results are expressed as the mean ± SD of at least three biological replicates. Statistical analysis was performed with SPSS 18.0, and comparisons between two groups were performed with one-way ANOVA followed by the LSD test. A *P* value less than 0.05 was considered to indicate statistical significance.

## 3. Results

### 3.1. Immunofluorescence Analysis of Human RPE Cells

**Figure 1:**
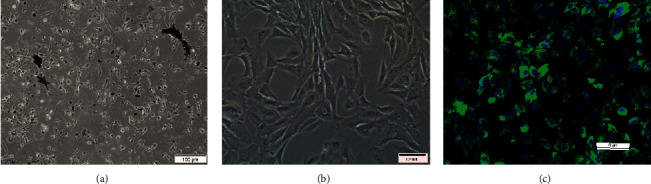
(a) Primary human RPE cells showing high levels of confluence and pigmentation after 24 (h). (b) Third-generation RPE cells are spindle-shaped, triangular, or irregular in shape, with a reduced number of cytoplasmic pigment granules. (c) Third-generation RPE cells fixed in 4% paraformaldehyde and incubated with an anti-RPE65 antibody. DAPI (blue) was also added to stain the nuclei.

The primary human RPE cells were spindle-shaped with many cytoplasmic pigment particles. The fourth-generation human RPE cells were polygonal or irregular in shape, and the number of cytoplasmic pigment granules was decreased. Immunofluorescence staining was used to characterize the expression of RPE-specific biomarkers, and the results showed green fluorescence in the RPE cells, indicating the expression of RPE65 (Figure 1).

### 3.2. Levels of Cytoplasmic Ca^2+^

**Figure 2:**
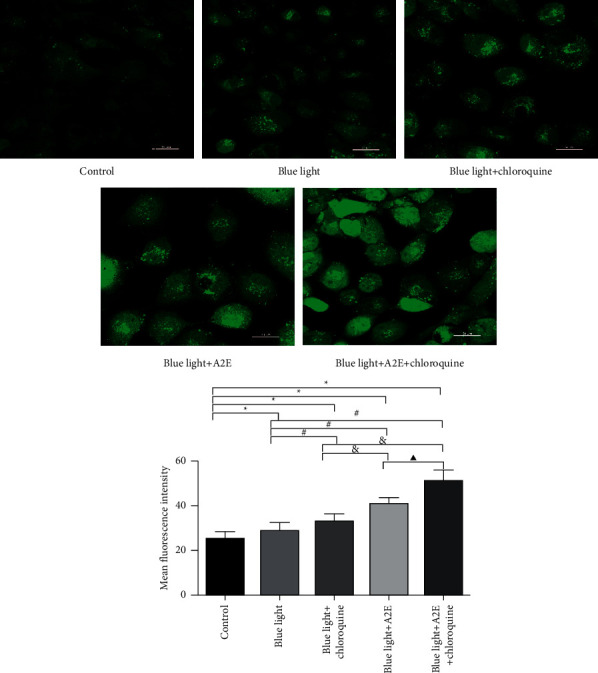
The cytoplasmic Ca^2+^ level. To determine the Ca^2+^ level in the cytoplasm, the fluorescence intensity of Ca^2+^ in RPE cells was assessed by confocal microscopy using the Ca^2+^-sensitive dye Fluo-3/AM. Scale bar = 20 *µ*m. The graph shows the quantified data. The data are presented as the mean ± SD (*n* = 3). An LSD test was conducted following one-way ANOVA. ^*∗*^*P* < 0.05 vs. control cells; ^#^*P* < 0.05 vs. blue light-treated cells; and ^&^*P* < 0.05 vs. blue light + chloroquine-treated cells; ^▲^*P* < 0.05 vs. blue light + A2E-treated cells.

Ca^2+^ in the cytoplasm was labeled with Fluo-3/AM, and the fluorescence intensity was measured using a laser scanning confocal microscope. We found that among that in other cells, the level of Ca^2+^ in the control cells was the lowest (*P* < 0.05). The Ca^2+^ level in the blue light + *A*2E + chloroquine-treated cells was higher than that in the other cells (*P* < 0.05). The increase in Ca^2+^ levels was more significant in the blue light + A2E-treated cells and blue light + chloroquine-treated cells than in the blue light-treated cells (*P* < 0.05) (Figure 2).

### 3.3. The Levels of IP3 and DAG in RPE Cells

**Figure 3:**
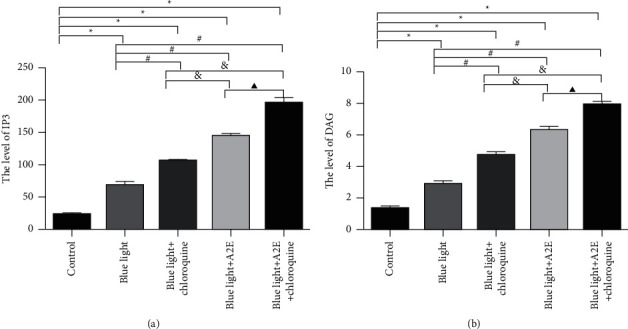
Intracellular IP3 and DAG levels in RPE cells. The intracellular levels of IP3 and DAG were measured by ELISA. The data are shown as the mean ± SD (*n* = 3). An LSD test following one-way ANOVA was performed. ^*∗*^*P* < 0.05 vs. control cells; ^#^*P* < 0.05 vs. blue light-treated cells; ^&^*P* < 0.05 vs. blue light + chloroquine-treated cells; ^▲^*P* < 0.05 vs. blue light + A2E-treated cells.

The intracellular levels of IP3 and DAG were measured by ELISA and found to be lower in control cells than in the other cells (*P* < 0.05). Compared with the other cells, the blue light + A2E + chloroquine-treated cells had the highest levels of IP3 and DAG (*P* < 0.05). The levels of IP3 and DAG in the blue light + A2E-treated cells and blue light + chloroquine-treated cells were higher than those in the blue light-treated cells (*P* < 0.05) (Figure 3).

### 3.4. PKC Activity in RPE Cells

**Figure 4:**
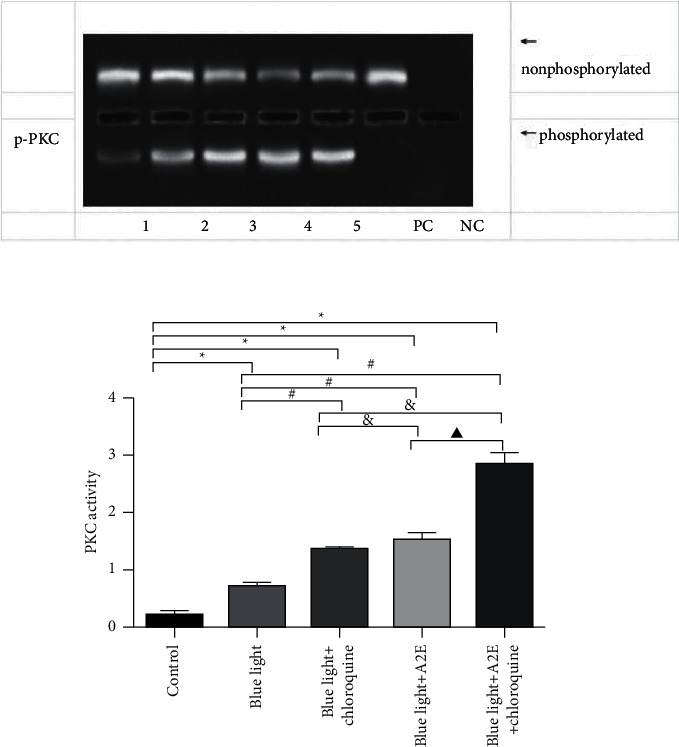
Changes in PKC activity in RPE cells, as shown by agarose gel electrophoresis. The ratio of the gray value of phosphorylated PKC and that of nonphosphorylated PKC indicated PKC activity. The data are presented as the mean ± SD (*n* = 3). An LSD test was conducted following one-way ANOVA. ^*∗*^*P* < 0.05 vs. control cells; ^#^*P* < 0.05 vs. blue light-treated cells; ^&^*P* < 0.05 vs. blue light + chloroquine-treated cells; ^▲^*P* < 0.05 vs. blue light + A2E-treated cells.

Intracellular PKC activity was measured with a nonradioactive nuclide assay, which showed that PKC activity was lower in the control group than in the other groups (*P* < 0.05). The blue light-treated cells exhibited decreased PKC activity compared with that in the blue light + A2E-treated cells and blue light + chloroquine-treated cells (*P* < 0.05). Blue light + *A*2E + chloroquine-treated cells exhibited the highest PKC activity (*P* < 0.05) (Figure 4).

## 4. Discussion

AMD is a progressive blinding disease with no cure and is the main cause of visual disability in industrialized countries [10–12]. Increasing evidence has shown that mitochondrial and RPE cells damage plays important roles in the pathogenesis of AMD [13]. RPE cells participate in phototransduction, and blue light is the most harmful factor to RPE cells [14]. RPE cells can also internalize the outer membrane disc of photoreceptors with the participation of CD36 and MerTK and begin to degrade these factors through the autophagy/lysosome pathway [15, 16], but undegraded residue accumulates in RPE cells in the form of lipofuscin, which leads to RPE cell senescence and apoptosis [17, 18]. A2E is the main fluorescent substance associated with lipofuscin. Through indirect photooxidation, A2E can damage lysosomes, increase lysosomal membrane permeability, and trigger inflammation [19, 20]. Therefore, we examined the effects of A2E on intracellular Ca^2+^, IP3, and DAG levels and PKC activity in the Ca^2+^-PKC signaling pathway during blue light irradiation.

Ca^2+^ is a second messenger that regulates various physiological and pathological functions, such as secretion, contraction, metabolism, gene transcription, and cell death [21]. Intracellular Ca^2+^ homeostasis is an important factor for maintaining normal cell function. Changes in intracellular Ca^2+^ concentrations determine cell survival or death. Close contact between the ER, plasma membrane, and other intracellular organelles participates in controlling Ca^2+^ homeostasis.

PKC regulates a variety of cellular processes by participating in the inositol phosphate signaling pathway [22]. All members of the PKC family are composed of an N-terminal regulatory region and a C-terminal catalytic domain connected by a protein-sensitive hinge zone. PKC exists in the cytoplasm and is generally inactive. This protein is water-soluble in its inactive state and free in the cytosol, but it becomes a membrane-bound enzyme after activation [23]. The activation of PKC is lipid-dependent and requires the presence of DAG, as well as an increase in the cytosolic Ca^2+^ concentration [24].

When A2E accumulates to a certain extent in the lysosomes of RPE cells, the phototoxicity of A2E increases the permeability of the lysosomal membrane [25], which releases a variety of acidic enzymes into the cytoplasm, inducing reactive oxygen species (ROS) generation and apoptosis [26, 27]. In this study, Ca^2+^ levels in blue light-treated cells and blue light + A2E-treated cells were higher than those in control cells, and Ca^2+^ levels in blue light + A2E-treated cells were higher than those in blue light-treated cells, indicating that blue light and A2E synergistically increased intracytoplasmic Ca^2+^ levels. After human RPE cells are exposed to A2E and/or blue light, these stimuli activate PLC through tyrosine protein kinase, stimulatory G proteins, or other pathways. PLC catalyzes the cleavage of phosphatidylinositol-4,5-bisphosphate (PIP2) in the membrane to produce IP3 and DAG. IP3 is water-soluble and binds the IP3 receptor on the endoplasmic reticulum membrane to release Ca^2+^ into the cytoplasm, resulting in an increase in intracellular Ca^2+^ levels. DAG is a fat-soluble molecule that reduces the level of Ca^2+^ required for PKC activation and synergizes with Ca^2+^ to activate PKC [28]. Cells undergo apoptosis in response to continuous exposure to high concentrations of free Ca^2+^ [29]. PKC can phosphorylate serine and threonine residues on various proteins and then promote cell proliferation and apoptosis [30, 31]. Our results showed that blue light and A2E increased cytoplasmic Ca^2+^ levels in human RPE cells and increased the activity of PKC, thereby inducing RPE cell apoptosis.

Chloroquine is a lipophilic weak base with a pH of approximately 7.4 that exists in three forms: unprotonated (CQ), singly protonated (CQ+), and diprotonated (CQ++) forms [32, 33]. Chloroquine, which is commonly used to prevent malaria, also inhibits autophagy by inhibiting the activity of lysosomal enzymes by changing the pH level [33]. Lysosomal inhibition can begin with the degradation of LAMP-2 in the glycocalyx, which decreases phagocytic uptake and increases exocytosis, leading lysosomal hydrolases to leak into the cytosol [34]. When lysosomal permeability increases or lysosomes rupture, released cathepsins B and D can cleave BCL-2 family proteins, which can cause mitochondrial dysfunction and activate the caspase-mediated apoptosis pathway [35, 36]. Chloroquine can also induce the accumulation of lipid bodies, which may contribute to drusen formation and the mechanism of AMD [34]. In this study, the levels of Ca^2+^, DAG, and IP3 and PKC activity in blue light + chloroquine-treated cells were higher than those in blue light-treated cells, and the levels in blue light + A2E + chloroquine-treated cells were higher than those in blue light + A2E-treated cells. After the addition of chloroquine, the pH of the acidic lysosomes in the cell changed, and lysosome homeostasis was destroyed. In vitro, chloroquine can destabilize lysosomal membranes and promote the release of lysosomal enzymes and other substances, such as A2E and Ca^2+^, within cells [37, 38]. As a result, Ca^2+^, IP3, and DAG levels in the cytoplasm increase, which activates PKC, and minor leakage can lead to caspase-dependent or caspase-independent apoptosis, while the massive translocation of lysosomal proteases can cause necrosis [37].

## 5. Conclusion

In the present study, we found that blue light and A2E could increase the levels of Ca^2+^, IP3, and DAG in human RPE cells and enhance the activity of PKC; furthermore, these factors were found to have a synergistic effect. Chloroquine further increased the levels of Ca^2+^, IP3, and DAG and PKC activity in human RPE cells exposed to blue light and/or loaded with A2E. However, our observations have some limitations. The blue light was a common denominator in our study of the additive effects of A2E and/or chloroquine. Since no A2E-only controls were used, the effect of A2E alone on the levels of Ca^2+^, IP3, and DAG and PKC activity could not be explored, and evidence that chloroquine increases lysosomal permeability is scarce, which requires further investigation.

## Data Availability

The data used to support the findings of this study are available from the corresponding author upon request.

## Abbreviations

A2E: N-Retinylidene-N-retinylethanolamine
RPE: Retinal pigment epithelial
PKC: Protein kinase C
IP3: Inositol triphosphate
DAG: Diacylglycerol.
